# Factors influencing emotional support of older adults living in the community: a scoping review protocol

**DOI:** 10.1186/s13643-023-02346-7

**Published:** 2023-10-04

**Authors:** Rashmi Devkota, Greta Cummings, Kathleen F. Hunter, Colleen Maxwell, Shovana Shrestha, Liz Dennett, Matthias Hoben

**Affiliations:** 1 Faculty of Nursing, College of Health Sciences, University of Alberta, Edmonton, AB Canada; 2 School of Pharmacy, University of Waterloo, Waterloo, ON Canada; 3 John W. Scott Health Sciences Library, University of Alberta, Edmonton, AB Canada; 4 School of Health Policy and Management, Faculty of Health, York University, Toronto, ON Canada

**Keywords:** Emotional support, Older adults, Community, Public health

## Abstract

**Background:**

Emotional support is key to improve older adults’ subjective health, and psychological, social and emotional well-being. However, many older adults living in the community lack emotional support, increasing the risk for loneliness, depression, anxiety, potentially avoidable healthcare use and costs, and premature death. Multiple intersecting factors may influence emotional support of older adults in the community, but these are poorly understood. Studies have focused on specific populations (e.g., older adults with depression, cancer). Although relevant, these studies may not capture modifiable factors for the wider and more diverse population of older adults living in the community. Our scoping review will address these important gaps. We will identify and synthesize the evidence on factors that influence emotional support of older adults in the community.

**Methods:**

We will use the Johanna Briggs Institute updated methodological guidance for the conduct of scoping reviews to guide our review process*.* We will search MEDLINE, EMBASE, APA Psycinfo, CINAHL, Dissertations and Theses Global, and Scopus from inception. We will include studies published in English, examining factors influencing emotional support of older adults residing in community, without restrictions on the study design or year of publication. We will also include gray literature (dissertations and reports). Two independent reviewers will conduct title, abstract, and full-text screening, as well as risk of bias assessment, using validated quality appraisal tools based on study designs. Discrepancies will be resolved by consensus. The primary reviewer will extract the data from all studies, and the second reviewer will check the extractions of all the studies. We will use descriptive statistics and narrative synthesis for analysis. Family/friend caregivers and older adults involved as an advisory group will help with explaining the findings in terms of whether associations observed reflect their experiences and reality. We will analyze the discussion and generate themes, and summarize in a narrative form.

**Discussion:**

This scoping review may identify factors that could be modified or mitigated to improve emotional support provision for older adults residing in community. The knowledge will inform the development of tailored interventions directed to older adults and their caregivers.

**Systematic review registration:**

https://doi.org/10.17605/OSF.IO/4TAEB (associated project link: osf.io/6y48t).

**Supplementary Information:**

The online version contains supplementary material available at 10.1186/s13643-023-02346-7.

## Background

Social support plays crucial role in older adults’ life (like younger individuals) [1–3]. Older adults who experience the death of friends or family members, functional and/or cognitive decline, reduced ability to attend social events, and loss of social contacts need support to cope with these losses [4–7]. Social support is referred as the interactive process in which person’s social networks (e.g., family, friends, neighbors, churches) or paid support services (e.g., physicians, nurses, therapists, and personal support workers) [8–13] provide assistance to the individual, including emotional (e.g., care, concern, love), material (e.g., services, goods, financial help, assistance), and informational (e.g., knowledge, advice) support [14, 15]. Emotional support, therefore, is one aspect of the broader concept social support.

Emotional support is crucial for the health and well-being of human beings (including older adults) [16]. Emotional support is defined as the expression of positive affect, empathetic understanding, and the encouragement of expressions of feelings, or the offering of advice, information, guidance or feedback [17]. It is a key process in close relationships, as well as an important determinant of satisfaction with these relationships [18]. Receipt of adequate emotional support is associated with improved subjective health [19], psychological, social and emotional well-being [20–23], and a reduced likelihood for depressive symptoms [24]. However, not all older adults receive sufficient emotional support. For example, between 20 and 48% older Atlantic Canadians (≥ 65 years) living in the community reported low emotional/informational support [25]. According to the Canadian Longitudinal Study on Aging (CLSA) [26], more than 17% of those aged 65 years and older felt like they did not have someone to confide in (measured as one of the sub-component of emotional/informational support) [26] which corresponds to almost 1.2 million older adults in Canada [27]. Lack of emotional support increases the risk of loneliness [28], anxiety [29], poor quality of life [28], and premature death [30].

Multiple intersecting factors may influence an older person’s emotional support. A body of evidence has identified common socio-demographic and health characteristics influencing older adults’ emotional support. Older adults who were widowed, divorced or were never married [7, 31, 32], those who had functional impairment [32], higher symptom burden [7], and those who lived alone [4] had lower levels of emotional support. Further, older adults with higher emotional support reported lower depression and chronic pain scores [31]. However, findings are inconsistent for increasing age, female sex, lower education, and lower income [6, 7, 31–34]. These inconsistencies may be attributed to the differences in sample characteristics and settings, and approaches to measuring emotional support across research studies. Research has also identified relationship-level factors that influence older adults’ emotional support. For instance, older adults with restricted social networks reported less emotional support [32], and those who had traumatic childhood experiences, such as emotional neglect, felt more emotionally isolated in old age [35]. Studies suggest that community-based support resources, such as the availability of community programs, are essential to develop and broaden the social network [36, 37]. The broader social networks provide opportunities to gain companionship and positive social interaction [32], and increase opportunities to receive various kind of support resources (e.g., instrumental, emotional) offered by their network members [38]. Further, racialized communities compared to white populations are reported to have higher levels of emotional and instrumental support [39–41]. However, these studies do not differentiate between emotional and instrumental support [39–41]. Age-related prejudices (ageism) promote social exclusion and limit social participation and thus lead to low social support [42, 43]. These studies do not indicate the types of social support measured [39–43]. Most studies on older adults’ emotional support have focused on specific populations, such as older adults with depression, cancer, diabetes, multiple illnesses, or disabilities [7, 44–47]. These studies are relevant. However, they may miss modifiable factors influencing emotional support for the wider and more diverse population of older adults.

Previous reviews have mainly focused on the influence of social support on morbidity, mortality, or healthcare service utilization [15, 48–51]. To the best of our knowledge, no review has comprehensively synthesized the available literature on factors that influence emotional support of older adults in general (rather than specific older adult populations), and that include all research designs. This scoping review will address this critical knowledge gap. With an aging population, maintaining older adults’ health, well-being and independence is a key public health priority around the globe [52–54]. To achieve this, a better understanding of factors influencing older adults’ emotional support is a prerequisite [16]. We will identify and synthesize the available evidence on factors that influence emotional support of older adults living in community.

## Theoretical guidance

**Fig. 1:**
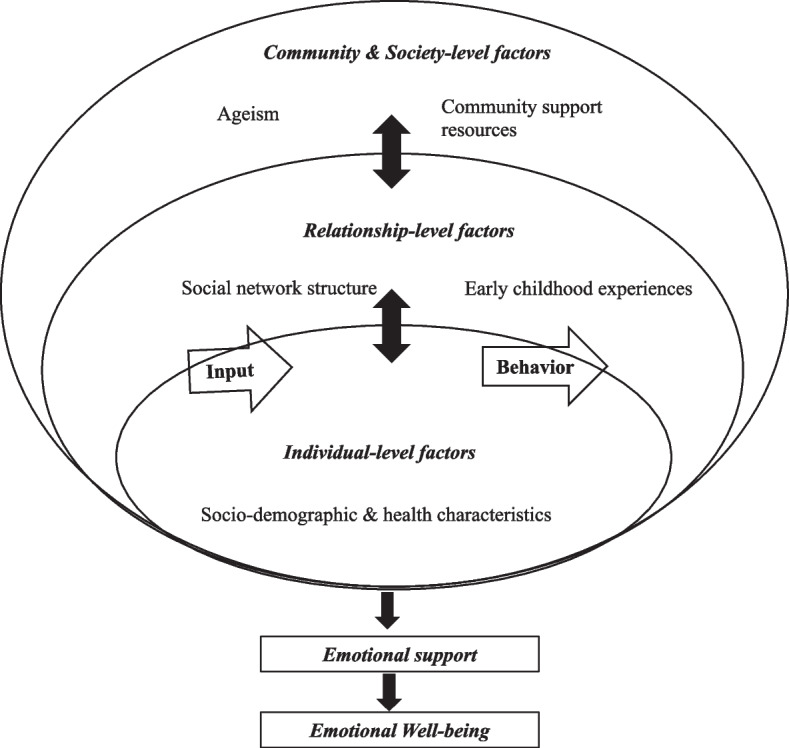
*Socio-ecological model. Adapted from *Bronfenbrenner, U. (1977) [57]. This model is inspired by Bronfenbrenner’s work on ecology of human development [57]. Thus, publication permission is not required (confirmed with American Psychologist Journal-the copyright holder of the original work)

We will use the social-ecological model (Fig. 1) to inform this review. The socio-ecological model is an adaptation of the earlier concepts from Bronfenbrenner’s ecological theory [55–57]. The social-ecological perspective assumes that human beings are a product of their thoughts, interpersonal relationships, organizational entities, and the community structures, systems, and policies which they are embedded in [58, 59]. Further, the culmination of these factors influences and predicts, or explains, health and well-being outcomes [58]. This social-ecological model posits that there are multiple factors at three levels that influences older adults’ emotional support: (1) Individual (e.g., race/ethnicity), (2) relationship (e.g., social network), and (3) community (e.g., availability of community programs and events) and society (e.g., ageism) levels [2, 55, 58, 60, 61]. The model explicates how factors at one level are embedded within or influence factors at another level, offering a multifaceted way to capture the complex interplay among individual, relationship, community, and society factors that influences older adults’ emotional support. Further, it posits that receipt of adequate emotional support influences older adults’ emotional well-being. Emotional support is important for individuals to lessen distress, cope more effectively with problems, and to maintain a positive self-concept and positive outlook on their life [16]. Further, it supports an individual to express positive emotions and regulate negative emotions [62–66]. This helps to attain a sense of balance between pleasant and unpleasant emotions [67–69]. Experiencing more pleasant than unpleasant emotions is central to a person’s emotional well-being [70, 71]. The levels and factors specified in socio-ecological model will guide inclusion or exclusion of studies (i.e., studies will be included if they assess any of the factors mentioned in the socio-ecological model) and data extraction on factors associated with emotional support of older adults. Details on this process is provided in Data extraction and Analyses section.

## Research question

What are the factors that influence emotional support of older adults living in community?

## Methods/design

This is a protocol of a scoping review that aims to systematically map the literature available on factors influencing older adults’ emotional support. We used scoping review as it allows to capture the diverse literature and study designs on this topic. The Johanna Briggs Institute updated methodological guidance for the conduct of scoping reviews will guide the processes of this scoping review [72]. To facilitate complete and transparent reporting of the review process, we will report the results of the synthesis according to the Preferred Reporting Items for Systematic Reviews and Meta-Analyses–Scoping Review (PRISMA-ScR) reporting guideline (Additional file 1) [73]. The review protocol follows the Preferred Reporting Items for Systematic Reviews and Meta-Analyses Protocols (PRISMA-P) 2015 statement (Additional file 2) [74]. We will involve people with lived experience (family/friend caregivers, and older adults) as an advisory group to further contextualize the review findings. Details are provided in Older adults and family engagement section.

### Inclusion and exclusion criteria

We will use broad inclusion/exclusion criteria (Appendix Table 1) to obtain relevant papers published in the English language without any restrictions on the study design or year of publication. We will not systematically search for grey literature; however, we will only include those grey literature (i.e., dissertations and reports) identified during the database search.

### Search strategy

A health sciences librarian (LD) developed searches for Medline (Ovid MEDLINE(R) ALL), Embase (Ovid interface), APA Psycinfo (Ovid Interface), CINAHL Plus with Full Text (EBSCOhost interface), Dissertations and Theses Global (Proquest Interface), and Scopus. These databases will be searched from the date of inception. The search combines an extensive list of terms for three concepts: emotional support AND family/friend caregivers or formal care providers AND older adults. Articles focused on nursing home and assisted living residents were removed from the search. Case reports and non-research articles were removed from the search where possible. No date, language, or study design limits were used. All the searches in six databases are included as an Additional file 3. We will conduct title/Abstract and full text screening in COVIDENCE. We will review the reference lists of included articles and reviews for additional studies.

### Study selection and screening

Two reviewers (RD and SS) will independently screen the titles and abstract of retrieved studies in batches, screening the first 100 articles [75] and then a consensus meeting follows to discuss issues and reconcile decisions. They will follow the same process for the remaining articles. Full-text screening of all references not definitely excluded in the previous step will follow a similar process. We will consult a third reviewer if two reviewers cannot agree upon the inclusion or exclusion of a study. We will use the PRISMA flow diagram (Appendix Fig. 2) to report the selection of articles during each phase with the rationale and to ensure the transparency of the selection of studies.

### Data extraction

We developed a data extraction form (Google Form, Additional file 4) to document details of eligible studies. Categories include: (1) study characteristics: year of publication and data collection, publication type (e.g., journal, thesis, reports), country of origin of the study, research aim/question, (2) theoretical framework, (3) design/methods: study design, setting, sample and sampling methods, data collection methods, outcomes and measures used, types of emotional support assessed and measures used, analyses applied, and (4) main findings: factors found to influence older adults’ emotional support—categorized based on their correspondence with the factor levels—individual (e.g., age, sex, marital status, race/ethnicity, chronic conditions), relationship (e.g., social network, early childhood experiences), or community (e.g., availability of community programs and events) and society (e.g., ageism) of the socio-ecological model. We will pre-test the data extraction form using five randomly selected studies to ensure feasibility of the data extraction form [76]. Two reviewers (RD and SS) will independently extract data from the 10% of the studies, followed by a consensus meeting to calibrate extraction. Reviewers will discuss any discrepancies, come to consensus, and the update the form (if needed) before extracting data from the remaining studies. RD will extract the data from all remaining studies, and SS will check the extractions of all the studies. Any discrepancies will be solved through discussion, and if needed, a third reviewer will help to resolve the differences.

### Quality appraisal

Quality appraisal is generally not recommended in scoping reviews [72]. However, we will conduct quality assessments to be able to speak to the quality of the studies and to increase the credibility of review findings. We will use five validated checklists to assess the methodological quality of the included studies after full text screening: (a) the Checklist for systematic reviews and research synthesis for systematic reviews, meta-analysis, scoping reviews, integrative reviews, and narrative reviews [77], (b) the Quality Assessment Tool for Quantitative Studies (QATQS) for randomized controlled trial (RCT), controlled trial (CT), pre-post [78, 79], (c) the Newcastle–Ottawa Scale (NOS) for any observational study (cohort study, cross-sectional study) [80], (d) the CASP for qualitative research studies [81], and (e) the Mixed Method Appraisal Tool (MMAT) for mixed method study designs [82, 83].

We will use the method developed by De Vet et al. [84] to score the overall quality of each study. For this, we will calculate the ratio of the obtained score to the maximum possible score (varies depending on the checklist used and the number of checklist items applicable) for each study (possible range 0–1). Each study will then be categorized as: weak (≤ 0.50), low moderate (0.51–0.66), high moderate (0.67–0.79), or strong (≥ 0.80). However, we will not exclude studies based on the methodological quality. RD and SS will independently appraise all the included studies. A consensus meeting will follow to solve any discrepancies after completing 10% of the appraisal of included studies (same process for the remaining appraisals). We will consult a third reviewer if needed to resolve any disagreements. We will report summary of quality of included studies. We will summarize and describe the key areas of strength and weakness for all studies within each type of research design.

### Analyses

We will descriptively present the number and proportion of studies that represent each category (e.g., country of origin, study setting, design, risk of bias category) using figures and tables. We will use descriptive statistics and narrative synthesis to summarize review findings [85]. In this process, we will identify and categorize the factors influencing older adults’ emotional support identified in each study based on their correspondence with individual, relationship, or community, and society level factors of socio-ecological framework. For quantitative results, we will report the number and proportion of studies reporting statistically significant positive and negative associations and non-significant associations (vote counting) by factor category as informed by socio-ecological framework. For qualitative results, we will conduct a content analysis of the key themes and factors assessed and we will indicate if the content of these themes varied across studies. We will report each theme to illustrate similarities and differences in their relationships with older adults’ emotional support. We will then categorize them into either individual, relationship, or community and society level factors according to how these themes align with either of the factor levels of socio-ecological framework.

For narrative synthesis, after organizing the review findings under three factor levels (individual, relationship, community/society) of socio-ecological framework, we will explore the relationships within (characteristics of individual studies and their reported findings) and between (between findings of different studies) included studies [85]. Socio-ecological framework will guide in understanding the relationships between different factors identified and older adults’ emotional support. Finally, we will assess the strength of the evidence (based on methodological quality of the included studies) for drawing conclusions about the influence of factors on older adults’ emotional support. Further, we will present the list of factors descriptively in table format. The analysis process will be iterative, and depend on the concrete references retrieved. Therefore, we will refine the synthesis strategies as needed and transparently document and report the process and any decisions made.

### Older adult and family engagement

We will involve family/friend caregivers from BC and Alberta, and older adults as an advisory group. While contacting advisory group members, we informed them about the objective of the review, their role in the research team, what is expected of them and their time commitments. We have presented a summary of the proposed review to this group in November 2021 and received their input on important issues (e.g., definition of emotional support) to guide the review. We will conduct one Zoom-based group discussion (approximately one hour in total) after data synthesis from the scoping review. Older adults and family members will assist with evaluating the findings. They will (a) help with explaining the patterns (e.g., associations)–if the associations reflect their experiences, if they agree or disagree with the findings, (b) assist in understanding if our findings include important gaps or if any additional focus is needed, and (c) provide their perspective on what can be done to emotionally support older adults in the community. We will obtain verbal informed consent from the respective patient-partners to participate in the focus group discussion. Two researchers will facilitate the recorded discussions. We will transcribe the focus group recordings verbatim. We will conduct thematic analysis. For this, two researchers will independently code texts, develop a preliminary coding categories (which we will discuss among the review team), discuss and reconcile coding categories, look for similarities and differences between the codes and then identify major themes and group them into similar themes [86]. We will present these findings in the results section separately in a narrative form [85]. We will integrate the advisors in planning and production (co-authorship, critical feedback) of the review output.

## Dissemination

We intend to disseminate the review findings through peer-reviewed publication, policy briefs (to Public Health Agency of Canada, Health Canada, provincial and regional governments, and provincial and regional health authorities) and prepare lay summaries and presentations of the findings for older adults and family/friend caregivers. We will present the findings at conference on geriatrics or health services and policy (e.g., Canadian Gerontological Nursing Association, Canadian Association on Gerontology, International Association of Gerontology and Geriatrics). The review will provide recommendations for future research areas, health policy, and care practice.

## Limitations

We are limited to studies published in English language and as a result, we might miss potential studies that are non-English. As we are not systematically searching for gray literature, we might miss out potential gray literature. Another limitation is that only primary reviewer will extract the data from all the studies. However, another independent reviewer will check the extraction of all the studies.

## Discussion

The scoping review is an appropriate and valid review approach to map a body of literature on factors that determine older adults’ emotional support. The review may aid in identifying factors that can be modified or mitigated (e.g., availability of community programs or events, ageism, caregiver distress) to improve emotional support provision for older adults. This knowledge will inform the development of tailored services and interventions directed to improve the older adults’ emotional support, as well as, minimize older adults’ unmet emotional support needs. The involvement of stakeholders in evaluating the findings will ensure that the findings of this study are grounded, and impactful. Further, this will also provide opportunity for dissemination of findings to target audiences (i.e., through the networks of family/friend caregivers and older adults who will be involved as an advisory group in the review).

Identifying and understanding older adults’ emotional support has become one of the urgent public health priorities considering the growth in the aging population. To develop an effective solution to meet older adults’ emotional support needs, it is important first to understand the factors that influence older adults’ emotional support. We hope this scoping review, when conducted rigorously and systematically, will provide trustworthy evidence that contributes to the understanding of older peoples’ emotional support. We acknowledge that the scoping review is an iterative process, and any changes made to the plan will be made as transparent as possible.

### Supplementary Information

**Additional file 1.** Preferred Reporting Items for Systematic reviews and Meta-Analyses extension for Scoping Reviews (PRISMA-ScR) Checklist.

**Additional file 2.** PRISMA-P 2015 Checklist.

**Additional file 3.** Preliminary search strategy.

**Additional file 4.** Data Extraction Form-Emotional support.

## Figures and Tables

**Fig. 2:**
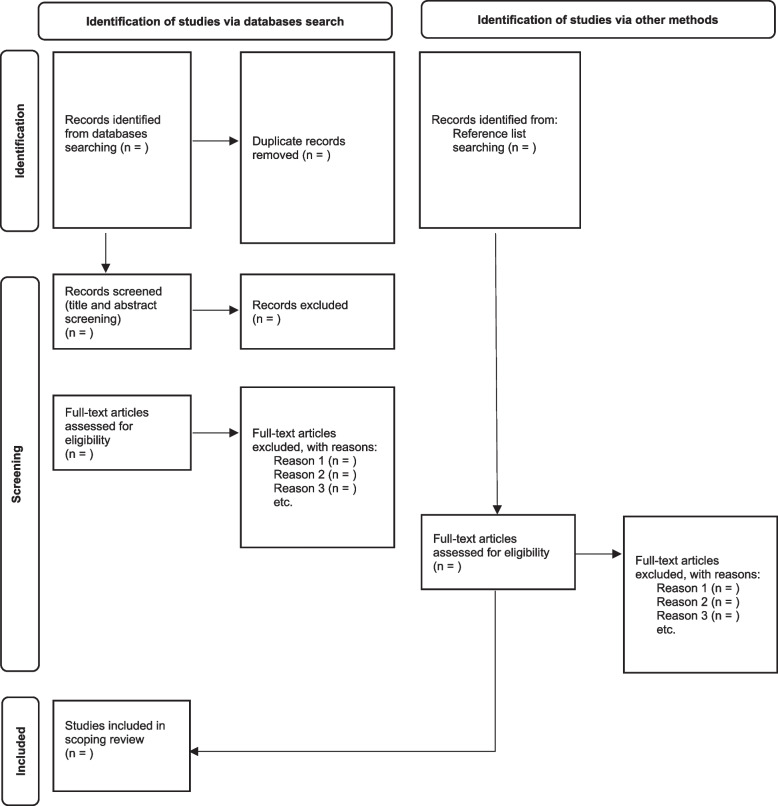
PRISMA flow diagram for article selection process

## Data Availability

We will include all data generated or analyzed in the published scoping review article. Upon request, other resources can be made available.

## Appendix

**Table 1:** Inclusion and exclusion criteria

	Inclusion criteria	Exclusion criteria
Study focus	• Studies examining factors that are associated with or influence older adults’ emotional support	• Studies only describing emotional support and/or assessing whether the emotional support needs are met but not assessing the association of any factors influencing older adults’ emotional support
Dependent variable	Emotional support, defined as a sole construct (expression of positive affect, empathetic understanding, and the encouragement of expressions of feelings) or in combination with informational support (offering of advice, information, guidance or feedback)	• Studies only assessing instrumental/tangible, positive social interaction, or affection social support without explicitly examining emotional support
Independent variables	• Socio-demographic and health characteristics of older adults (e.g., age, sex, marital status, race/ethnicity, chronic conditions) • Relationship factors (e.g., social network, early childhood experiences) • Community factors (e.g., availability of community programs and events) • Society related factors (e.g., ageism)	
Study design	• Primary empirical quantitative studies regardless of the research design (e.g., randomized controlled trials, non-randomized trials with or without a control group, cohort studies, cross-sectional studies, time series or regression discontinuity designs) • Qualitative studies (e.g., qualitative interviews, focus groups, ethnographic observations, qualitative case studies) • Mixed-methods studies	• Research protocols, case reports, editorials, opinion papers, comments, newspaper articles, historical articles, lecture notes, presentations, personal narratives
Setting	• Older adult’s private home, apartment, rented home	• Studies focusing on nursing homes (residential long-term care), daycare/centers, rehabilitation centers, assisted living settings, hospitals
Population	• Studies that only include older adults age ≥ 65 •Studies that include older adults (≥ 65) and younger adults as long as results are reported separately for older adults	• Studies including no participants aged ≥ 65 years • Studies including older adults but not reporting results specific to this sub-group
