# Supplementary material for: Factors influencing emotional support of older adults living in the community: a scoping review protocol
Source: Syst Rev. 2023 Oct 4;12:186. doi: 10.1186/s13643-023-02346-7 (PMC10548654; doi:10.1186/s13643-023-02346-7)
Supplement: Supplementary file 3 — Additional file 3. Preliminary search strategy. [file 13643_2023_2346_MOESM3_ESM.docx]

**Additional file 3:** Preliminary search strategy

**Ovid MEDLINE(R) ALL 1946 to July 12, 2022**

Date searched: July 13, 2022

Results: 2155

1.(medical outcome study social support or interpersonal support evaluation list or (emotion* and (social support adj2 (rating or scale or score or questionnaire)))).mp.

2.((emotion* adj4 (need or needs or requir*)) and (support* or comfort* or relationships or interactions or connections)).mp.

3.((emotional adj3 support*) or emotional experience*).mp.

4.1 or 2 or 3

5.((family not family-histor*) or families or social network or social networks or spouse* or partner* or wife or wives or husband* or adult child* or caregiver* or care giver* or neighbour* or neighbor* or friend or friends or grandchild* or close-tie* or weak-tie* or network-tie* or nurse* or physician* or doctor* or clinician* or general practitioner* or therapist* or care aide* or nursing assistant* or home care or home support services or home health care or home healthcare).mp.

6.exp Aged/ or ("over 65" or "65 and over" or "65 or over" or "65+ years" or retired or old age or older adult* or mature adult* or older person* or older individual* or mature individual* or older people or aging or ageing or senior* or elder* or old* population* or aged population or older men or older women).mp.

7.exp nursing homes/ or institutionalization/ or hospitalization/ or inpatients/

8.("nursing home*" or "old age home*" or "care home*" or hospitalised or hospitalized or institutionali* or supportive living or assisted living).mp.

9.independent living/ or (non-hospitali* or non-institutionali* or senior* cent* or community or isolated or ((independent* or family or families) adj3 (living or dwelling)) or "at home" or "own home" or home-dwelling or home-visit* or home care or live* alone or living alone or "live on their own" or ("live* with" adj3 famil*) or home-based or ((age or aging or ageing) adj3 (place or home))).mp.

10.(7 or 8) not 9

11.(4 and 5 and 6) not 10

12.limit 11 to (case reports or editorial or historical article or lecture or news or newspaper article)

13.11 not 12

**Embase 1974 to 2022 July 12 (OVID Interface)**

Date searched: July 13, 2022

Results: 2414

Search saved as: "Emotional support of community seniors - Embase - July 2022"

1. (medical outcome study social support or interpersonal support evaluation list or (emotion* and (social support adj2 (rating or scale or score or questionnaire)))).mp.

2. ((emotion* adj4 (need or needs or requir*)) and (support* or comfort* or relationships or interactions or connections)).mp.

3. ((emotional adj3 support*) or emotional experience*).mp.

4. or/1-3

5. ((family not family-histor*) or families or social network or social networks or spouse* or partner* or wife or wives or husband* or adult child* or caregiver* or care giver* or neighbour* or neighbor* or friend or friends or grandchild* or close-tie* or weak-tie* or network-tie* or nurse* or physician* or doctor* or clinician* or general practitioner* or therapist* or care aide* or nursing assistant* or home care or home support services or home health care or home healthcare).mp.

6. Aged/ or exp Geriatrics/ or Gerontology/ or Geriatric psychotherapy/ or exp Geropsychology/ or frail elderly/ or very elderly/ or elderly care/ or geriatric care/ or aging/ or healthy aging/ or attitude to aging/

7. ("over 65" or "65 and over" or "65 or over" or "65+ years" or retired or old age or older adult* or mature adult* or older person* or older individual* or mature individual* or older people or senior* or elder* or old* population* or aged population or older men or older women).mp. or (aging or ageing).tw,kf.

8. aged hospital patient/ or institutionalized elderly/ or geriatric nursing/ or home for the aged/ or nursing home/ or nursing home patient/ or residential care/ or residential home/ or institutional care/ or institutionalization/ or ("nursing home*" or "homes for the ag*" or "old age home*" or "care home*" or "residential care" or "residential facilit*" or "residential home*" or hospitalised or hospitalized or institutionali* or ((senior* or retirement) adj2 (home or facilit* or residence)) or supportive living or assisted living).mp.

9. Independent Living/ or community dwelling person/ or (non-hospitali* or non-institutionali* or senior* cent* or community or isolated or ((independent* or family or families) adj3 (living or dwelling)) or "at home" or "own home" or home-dwelling or home-visit* or home care or live* alone or living alone or "live on their own" or ("live* with" adj3 famil*) or home-based or ((age or aging or ageing) adj3 (place or home))).mp.

10. (4 and 5 and (6 or 7)) not (8 not 9)

11. limit 10 to editorial

12. (10 and (Case report/ or (case-stud* or case-report*).jx. or (case-study or (case-report not case-report-form*)).mp.)) not (case-series or case-control).mp.

13. 10 not (11 or 12)

**APA PsycInfo 1806 to July Week 2 2022   (OVID Interface)**

Date searched: July 13, 2022

Results: 2013

Search saved as:Emotional support of community seniors - Psycinfo - July 2022

1. (medical outcome study social support or interpersonal support evaluation list or (emotion* and (social support adj2 (rating or scale or score or questionnaire)))).mp.

2. ((emotion* adj4 (need or needs or requir*)) and (support* or comfort* or relationships or interactions or connections)).mp.

3. ((emotional adj3 support*) or emotional experience*).mp.

4. or/1-3

5. ((family not family-histor*) or families or social network or social networks or spouse* or partner* or wife or wives or husband* or adult child* or caregiver* or care giver* or neighbour* or neighbor* or friend or friends or grandchild* or close-tie* or weak-tie* or network-tie* or nurse* or physician* or doctor* or clinician* or general practitioner* or therapist* or care aide* or nursing assistant* or home care or home support services or home health care or home healthcare).mp.

6. 4 and 5

7. limit 6 to ("380 aged " or "390 very old ")

8. geriatrics/

9. ("over 65" or "65 and over" or "65 or over" or "65+ years" or retired or old age or older adult* or mature adult* or older person* or older individual* or mature individual* or older people or aging or ageing or senior* or elder* or old* population* or aged population or older men or older women).mp.

10. 7 or 8 or 9

11. Residential care institutions/ or Nursing homes/ or institutionalization/ or ("nursing home*" or "homes for the ag*" or "old age home*" or "care home*" or "residential care" or "residential facilit*" or "residential home*" or hospitalised or hospitalized or institutionali* or ((senior* or retirement) adj2 (home or facilit* or residence)) or supported living or supportive living or assistive living or assisted living).mp.

12. Living alone/ or aging in place/ or retirement communities/ or (non-hospitali* or non-institutionali* or senior* cent* or community or isolated or ((independent* or family or families) adj3 (living or dwelling)) or "at home" or "own home" or home-dwelling or home-visit* or home care or live* alone or living alone or "live on their own" or ("live* with" adj3 famil*) or home-based or ((age or aging or ageing) adj3 (place or home))).mp.

13. (6 and 10) not (11 not 12)

14. (case reports/ or (case-stud* or case-report*).jx. or (case-study or case-report).mp.) not (case-series or case-control).mp.

15. 13 not 14

16. limit 15 to ("column/opinion" or editorial or encyclopedia entry or interview or obituary or review-book or review-media or review-software & other)

17. 15 not 16

**CINAHL Plus with Full Text  (EBSCOhost Interface)**

Date searched: July 13, 2022

Results:1839

Deselect: Apply equivalent subject terms

Search saved as: Emotional support of community seniors CINAHL July 2022 in ldennett's EBSCO account

S1. medical-outcome-study-social-support or interpersonal-support-evaluation-list or (emotion* and (social-support N2 (rating or scale or score or questionnaire))) OR ((emotion* N4 (need or needs or requir*)) and (support* or comfort* or relationships or interactions or connections)) OR (emotional N3 (support*)) or emotional-experience*

S2. (family not family-histor*) or families or "social network" or "social networks" or spouse* or partner* or wife or wives or husband* or adult-child* or caregiver* or care-giver* or neighbour* or neighbor* or friend or friends or grandchild* or close-tie* or weak-tie* or network-tie* or nurse* or physician* or doctor* or clinician* or general-practitioner* or therapist* or care-aide* or nursing-assistant* or home-care or home-support-services or home-health-care or home-healthcare

S3 (MH "Aged") OR (MH "Aged, 80 and Over+") OR (MH "Frail Elderly") OR (MH "Geriatrics") OR ("over 65" or "65 and over" or "65 or over" or "65+ years" or retired or old-age or older-adult* or mature-adult* or older-person* or older-individual* or mature-individual* or older-people or aging or ageing or grandparent* or grandmother* or grandfather* or senior* or elder* or old*-population* or aged-population or older-men or older-women)

S4 (MH "Residential Facilities") OR (MH "Nursing Homes+") OR (MH "Institutionalization") OR (MH "Hospitalization") OR (MH "Aged, Hospitalized") OR (MH "Nursing Home Patients") OR nursing-home* or "homes for the ag*" or "old age home*" or "care home*" or "residential care" or "residential facilit*" or "residential home*" or hospitalised or hospitalized or institutionali* or ((senior* or retirement) N2 (home or facilit* or residence)) or supportive-living or supported-living or assisted-living or assistive-living

S5 (MH "Community Living") or non-hospitali* or non-institutionali* or senior*-cent* or community or isolated or ((independent* or family or families) N3 (living or dwelling)) or "at home" or "own home" or home-dwelling or home-visit* or home-care or live*-alone or living-alone or "live on their own"  or ("live* with" N3 famil*) or home-based or ((age or aging or ageing) N3 (place or home))

S6    S1 AND S2 AND S3 NOT (S4 NOT S5)

S7. ((MH "Case Studies") OR SO(case-stud* or case-report*)  OR ( case-study or case-report ) ) NOT (case-series or case-control)

S8:  S6 NOT S7

**Scopus (Advanced search)**

Date searched: July 13, 2022

Results:2108

( ( ( TITLE-ABS-KEY ( medical-outcome-study-social-support  OR  interpersonal-support-evaluation-list  OR  emotional-experience* )  OR  TITLE-ABS-KEY ( emotion*  AND  ( social-support  W/2  ( rating  OR  scale  OR  score  OR  questionnaire ) ) )  OR  TITLE-ABS-KEY ( ( emotion*  W/4  ( need  OR  needs  OR  requir* ) )  AND  ( support*  OR  comfort*  OR  relationships  OR  interactions  OR  connections ) )  OR  TITLE-ABS-KEY ( emotional  W/3  ( support* ) ) )  AND  TITLE-ABS-KEY ( ( family  AND NOT  family-histor* )  OR  families  OR  {social network}  OR  {social networks}  OR  spouse*  OR  partner*  OR  wife  OR  wives  OR  husband*  OR  adult-child*  OR  caregiver*  OR  care-giver*  OR  neighbour*  OR  neighbor*  OR  friend  OR  friends  OR  grandchild*  OR  close-tie*  OR  weak-tie*  OR  network-tie*  OR  nurse*  OR  physician*  OR  doctor*  OR  clinician*  OR  general-practitioner*  OR  therapist*  OR  care-aide*  OR  nursing-assistant*  OR  home-care  OR  home-support-services  OR  home-health-care  OR  home-healthcare )  AND  ( ( KEY ( aged )  AND NOT  KEY ( {Middle aged} ) )  OR  TITLE-ABS-KEY ( {over 65}  OR  {65 and over}  OR  {65 or over}  OR  {65+ years}  OR  retired  OR  old-age  OR  older-adult*  OR  mature-adult*  OR  older-person*  OR  older-individual*  OR  mature-individual*  OR  older-people  OR  aging  OR  ageing  OR  senior*  OR  elder*  OR  old*-population*  OR  aged-population  OR  older-men  OR  older-women ) ) )  AND NOT  ( TITLE-ABS-KEY ( nursing-home*  OR  "homes for the ag*"  OR  "old age home*"  OR  "care home*"  OR  "residential care"  OR  "residential facilit*"  OR  "residential home*"  OR  hospitalised  OR  hospitalized  OR  institutionali*  OR  ( ( senior*  OR  retirement )  W/2  ( home  OR  facilit*  OR  residence ) )  OR  supportive-living  OR  supported-living  OR  assisted-living  OR  assistive-living )  AND NOT  TITLE-ABS-KEY ( non-hospitali*  OR  non-institutionali*  OR  senior*-cent*  OR  community  OR  isolated  OR  ( ( independent*  OR  family  OR  families )  W/3  ( living  OR  dwelling ) )  OR  {at home}  OR  {own home}  OR  home-dwelling  OR  home-visit*  OR  home-care  OR  live*-alone  OR  living-alone  OR  {live on their own}  OR  home-based  OR  ( ( age  OR  aging  OR  ageing )  W/3  ( place  OR  home ) ) ) ) )  AND NOT  ( ( SRCTITLE ( case-stud*  OR  case-report* )  OR  TITLE-ABS-KEY ( {case study}  OR  {case report} ) )  AND NOT  TITLE-ABS-KEY ( {case-series}  OR  {case-control} ) )

**Dissertations and Theses Global  (Proquest database)**

Date searched: July 13, 2022

Results:414

((noft(medical-outcome-study-social-support or interpersonal-support-evaluation-list or emotional-experience*) or noft(emotion* and (social-support NEAR/2 (rating or scale or score or questionnaire))) OR noft((emotion* NEAR/4 (need or needs or requir*)) and (support* or comfort* or relationships or interactions or connections)) OR noft(emotional NEAR/3 (support*))) AND noft((family NOT family-histor*) or families or "social network" or "social networks" or spouse or partner or wife or wives or husband or adult-child or caregiver or care-giver or neighbour or neighbor or friend or friends or grandchild* or close-tie or weak-tie or network-tie OR  nurse  OR  physician  OR  doctor  OR  clinician  OR  general-practitioner  OR  therapist  OR  care-aide  OR  nursing-assistant  OR  home-care  OR  home-support-services  OR  home-health-care  OR  home-healthcare) AND noft( "over 65" OR "65 and over" OR "65 or over" OR "65+ years" OR retired OR old-age OR older-adult* OR mature-adult* OR older-person* OR older-individual* OR mature-individual* OR older-people OR aging OR ageing OR senior* OR elder* OR old*-population* OR aged-population OR older-men OR older-women)) NOT noft((nursing-home* OR "homes for the ag*" OR "old age home*" OR "care home*" OR "residential care" OR "residential facilit*" OR "residential home*" OR hospitalised OR hospitalized OR institutionali* OR ((senior* OR retirement) NEAR/2 (home OR facilit* OR residence)) OR  supportive-living  OR  supported-living  OR  assisted-living  OR  assistive-living) NOT (non-hospitali* OR non-institutionali* OR senior*-cent* OR community OR isolated OR ((independent* OR family OR families) NEAR/3 (living OR dwelling)) OR "at home" OR "own home" OR home-dwelling OR home-visit* OR home-care OR live*-alone OR living-alone OR "live on their own" OR ("live* with" NEAR/3 famil*) OR home-based OR ((age OR aging OR ageing) NEAR/3 (place OR home))))
