# Supplementary material for: Factors influencing emotional support of older adults living in the community: a scoping review protocol
Source: Syst Rev. 2023 Oct 4;12:186. doi: 10.1186/s13643-023-02346-7 (PMC10548654; doi:10.1186/s13643-023-02346-7)
Supplement: Supplementary file 4 — Additional file 4. Data Extraction Form-Emotional support. [file 13643_2023_2346_MOESM4_ESM.pdf]

Data extraction form_Emotional support

Data Extraction Form-Emotional support

Study characteristics

1. Initial of person entering data (including middle names, if applicable)
   [Examples are: Rashmi Devkota= RD]

2. First Author
   [Enter as: last name, first name]

3. Year of publication and data collection

4. Publication type
   [Examples: Journal, thesis, reports. For journals, state name of the journal]

5. Country of origin of study

6. Title of the study

[Copy paste the title from the article]

7. Research aim/question

[Extract the author's stated primary and secondary purposes]

8. Theoretical framework

[Just the name of the theoretical framework. No further details required]

Study design

Study design

9. Select the applicable study design

Mark only one oval.

- RCT *Skip to question 12*
- Controlled trial *Skip to question 12*
- Pre-Post *Skip to question 12*
- Cohort *Skip to question 12*
- Case-control *Skip to question 12*
- Cross-sectional *Skip to question 12*
- Qualitative *Skip to question 12*
- Mixed *Skip to question 10*
- Others *Skip to question 11*

Specify mixed design

10. If mixed design, select the most appropriate designs from the following list

Mark only one oval.

- Exploratory sequential design-study begins with qualitative data collection methods followed by quantitative methods
- Explanatory sequential design - study begins with quantitative methods followed by qualitative methods
- Concurrent- both quantitative and qualitative methods are conducted in parallel

Skip to question 12

Specify other types of designs

11. If other designs, provide descriptions

____

Methods of data collection

12. Method of data collection-Quantitative

Check all that apply.

- [ ] Participant completed survey/questionnaire
- [ ] Researcher completed survey/questionnaire with participant/structured participant intervie
- [ ] Administrative data review/structured chart review
- [ ] N/A
- [ ] Other: ______________

13. Method of data collection-Qualitative

Check all that apply.

- [ ] Semi-structured interview
- [ ] Focus group discussions
- [ ] N/A
- [ ] Other: ______________

Settings

[Refers to the settings from which participants were recruited from

14. Setting reported in the study

____

Sample and sampling methods

15. Sample size

16. Sampling method used
[e.g., random sampling, purposive sampling, stratified sampling]

17. Age
[Extract the average (mean or median) age, the percentage of older adults in different age categories identified in the article. If the study includes both young and older population, report t age of older adults only]

18. Sex

[Extract the percentage of the sample that was female/women and/or male/men. If the study includes both young and older population, report sex of older adults only]

Outcomes and measures used

19. Outcomes reported in the study and the measures used to assess those outcomes

[E.g., outcomes=emotional/informational support, measures used=MOS-SSS scale. Write the ful form of the scale or instrument used]

20. Types of emotional support assessed and measures used

Analysis applied-Quantitative

21. Analysis-Quantitative
[Specify the analysis. No description needed]

Analysis applied-Qualitative

22. Analysis-Qualitative
[Specify the analysis. No description needed]

Analysis applied-Mixed method

23. Analysis-Mixed method
[Specify the analysis. No description needed]

Main findings

24. Findings

[Extract the factors that are found to influence older adults' emotional support under three differ categories: individual (e.g., age, sex, race/ethnicity), relationship (e.g., social network), communi and society (e.g.., availability of community support group, ageism). For quantitative studies, rep the statistically significant positive and negative associations and non-significant associations. qualitative studies, key themes and factors assessed]

This content is neither created nor endorsed by Google.

Google Forms
